# Changes in Mental and Physical Health Outcomes Following One Day a Week Cardiopulmonary Rehabilitation in Regional New South Wales

**DOI:** 10.1111/ajr.70033

**Published:** 2025-03-24

**Authors:** Nnamdi Mgbemena, Jane Thompson, Uchechukwu Levi Osuagwu

**Affiliations:** ^1^ Discipline of Physiotherapy Charles Sturt University, Orange New South Wales Australia; ^2^ Cardiopulmonary Rehabilitation Unit Bathurst Health Service, Bathurst New South Wales Australia; ^3^ Bathurst Rural Clinical School (BRCS), School of Medicine Western Sydney University Bathurst NSW Australia

**Keywords:** cardiorespiratory, improvements, mental health, physical function, rehabilitation

## Abstract

**Introduction:**

Cardiopulmonary rehabilitation participation rates in regional Australia remain poor, with outcomes further worsened by the limited number of cardiopulmonary rehabilitation professionals in these settings.

**Objective:**

This study investigated the role of cardiopulmonary rehabilitation in improving physical and mental health outcomes of participants with heart or lung diseases in a regional NSW centre.

**Design:**

A retrospective study of adults who attended a 1‐h session per week cardiac or pulmonary rehabilitation programme at Bathurst Hospital between January 2021 and December 2023.

**Main Outcome Measures:**

Pre‐ and post‐rehabilitation assessments were conducted, which included heart rate, blood pressure, oxygen saturation, waist circumference, rating of perceived exertion, 5‐sit‐to‐stand test (5‐STS), 6‐min walk test (6MWT), and the patient health questionnaire‐9 for assessment of depression (PHQ‐9 score ≥ 10 = major depression).

**Findings:**

Data for eligible participants (*n* = 186, mostly males 57.5%), aged 69 ± 12 years, were analysed. There were statistically significant improvements (pre vs. post) in mean PHQ‐9 scores (6.3 vs. 4.2, *p* < 0.001), 5‐STS (15.8 vs. 12.5 s, *p* < 0.001), 6MWT (328.6 vs. 377.9 m, *p* < 0.001) and waist circumference (104.7 vs. 103.9 cm, *p* < 0.03) post‐rehabilitation. Compared with pre‐rehabilitation measures, the overall proportion with major depression was significantly lower by 50% (25.3% vs. 12.4%, *p* < 0.05) post‐rehabilitation. This decrease was significant for the cardiac (11.6% decrease) and pulmonary (15.4% decrease) rehabilitation participants.

**Conclusion:**

Despite the limiting structure of one session per week for the cardiopulmonary rehabilitation programme at this regional centre, participants showed significant improvements in their mental and physical health at the end of the programme. Funding such organic programmes will yield a greater positive impact on the health of people in this region.

**Summary**

- What is already known on this subject?
  ○ Attending cardiac or pulmonary rehabilitation has been shown to reduce mortality rates and hospital readmissions; however, there is limited information on the impact of these programmes in rural and remote Australia.
  ○ There are still poor cardiac and pulmonary rehabilitation participation rates in Australia, which necessitate the need for greater awareness of the benefits of these programmes, especially in rural and remote Australia.
- What this study adds?
  ○ This study contributes to the growing body of evidence supporting the benefits of cardiac and pulmonary rehabilitation programmes in improving health outcomes for individuals living with cardiovascular and respiratory conditions.
  ○ Attending even one session per week of cardiac or pulmonary rehabilitation can yield positive outcomes for participants, particularly in settings where resources are scarce.
  ○ Investing in and prioritising the dissemination of rehabilitation services in low resource areas in Australia becomes essential step toward fostering equitable access to quality healthcare for all.

## 1 Introduction

Cardiopulmonary rehabilitation is a multi‐component intervention offered to patients with heart or lung disease, which helps to improve their functional capacity and quality of life and reduce symptoms of dyspnoea and fatigue [1, 2]. In clinical practice, cardiopulmonary rehabilitation is designed as two independent programmes that are personalised to the participant's needs, where cardiac rehabilitation (CR) is run for 6–10 weeks, while pulmonary rehabilitation (PR) runs for 8 weeks [3, 4].

Introduced in the late 1960s, CR has greatly evolved over the last two to three decades. The programme has developed a large evidence base, the majority suggesting that comprehensive CR should also be targeted at participants with acute coronary syndrome (ACS), percutaneous coronary intervention, or who have undergone cardiac surgeries (coronary artery bypass graft and valvular replacement or repair) [5]. In Australia, existing data showed that 92,400 adults were admitted to the hospital on account of ACS between 2020 and 2021, with an estimated annual mortality rate of 12% [6, 7]. A recent study conducted in New South Wales, Australia, highlighted an increased incidence of emergency presentations and hospital readmissions for ACS among those living in the Hunter New England region compared to the metropolitan areas, reflecting the existing reality of poorer and inadequate specialised health care services in rural settings [8]. Despite the robust evidence of CR in reducing mortality rates and hospital readmissions, there is still a poor CR estimated participation rate of 30% in Australia [9]. Studies have attributed this low uptake in Australia to poor referral rates, poor access to services and adherence, and suboptimal sustainability [10].

Similarly, PR, which involves structured, multidisciplinary education, supervised exercises and psychosocial support, was originally targeted at patients diagnosed with chronic obstructive pulmonary disease (COPD), with recent clinical recommendations to offer this programme to patients with bronchiectasis, interstitial lung disease and pulmonary hypertension [2]. In Australia, approximately 53,000 adults aged 45 and above were hospitalised on a account of COPD between 2021 and 2022, with a mortality rate of 29.6 per 100,000 people in 2022 [11]. The prevalence of COPD is higher in rural and remote areas (3.9%) compared to urban settings (2.2%), with socio‐economic factors being a significant determinant of this disparity. Consequently, rural areas report elevated rates of both hospital admissions and mortality due to COPD compared to metropolitan areas [11]. Despite the demonstrated evidence of the positive effects of PR, poor participation (< 1.2%) remains a common finding in developed countries (Australia, New Zealand, Canada, Ireland, Sweden, the United Kingdom and the United States) [12]. In Australia, a prospective study reported that one‐third of patients with acute exacerbated COPD were referred to PR [13], while less than one‐fifth of the patients with stable COPD attended and completed at least half of the PR programme [14]. Also, prior studies among medical practitioners identified barriers to referral and adherence to PR, including poor knowledge of PR for the COPD population, unclear understanding of the referral process, access or travel difficulties and not being sure of the need to do more to achieve a behaviour change [15, 16].

Considering that heart disease and COPD remain the first and third leading causes of death globally, respectively, and the expected poorer participation rates in cardiopulmonary rehabilitation in regional and rural areas [17, 18, 19], there is still a need to reiterate the importance of these programmes in improving the physical and mental health outcomes of people living in regional and rural Australia. Therefore, this study was designed to demonstrate the role of cardiopulmonary rehabilitation in improving physical and mental health outcomes in patients with heart or lung diseases. It is expected that a significant improvement in health outcomes in these individuals would encourage participation in cardiopulmonary rehabilitation in adults living in these areas, where there remains a dearth of information on the impact of these programmes.

## 2 Methods

### 2.1 Study Design and Setting

This was a retrospective study of participants who attended the Cardiopulmonary rehabilitation centre at Bathurst Hospital, New South Wales, between January 2021 and December 2023. Participants were either referred to the CR or PR programme by health professionals or were self‐referred. Pre‐rehabilitation assessments were conducted by a nurse or physiotherapist at week 0, while post‐rehabilitation assessments were performed by the same health professional on the final day of week 6 for the CR programme and week 8 for the PR programme, based on the programme's current staffing availability.

### 2.2 Ethical Consideration

The study followed the tenets of the Declaration of Helsinki for human subjects (as revised in Brazil 2013). Prior to data collection, ethics approval for the study was obtained from the Western New South Wales Local Health District; Greater Western Human Research Ethics Committee, University Research Ethics Committee (2021/ETH00556). All participants provided written informed consent before data collection for the use of their data for research purposes.

### 2.3 Inclusion and Exclusion Criteria

Participants were included in this study if they were adults (aged 18 years and above), completed their rehabilitation programme in Bathurst Hopsital Centre within the study duration and had pre‐ and post‐rehabilitation data for the measured outcomes recorded in their hospital records. Those with incomplete demographic and/or clinical data for the studied variables were excluded.

### 2.4 Data Collection

Participants' socio‐demographic data including age, sex, height, weight and referring condition/reason were retrieved from their electronic medical records by the nursing team. Physical measures (heart rate, blood pressure, oxygen saturation levels, waist circumference, handgrip strength [HGS], rating of perceived exertion, 5‐sit‐to‐stand test [5‐STS], 6‐min walk test [6MWT]) and mental health measures (patient health questionnaire‐9, PHQ‐9) were obtained before and after they completed the rehabilitation programme. These assessments were done under the essential and desirable cardiac rehabilitation best practice statements on the content to be delivered in phase II CR programme [20]. Participants' blood pressure, heart rate and oxygen saturation testing were done in this order, followed by 6MWT, HGS testing in standing, 5‐STS, waist circumference and filling out the PHQ‐9. Participants' baseline perceived exertion was assessed using the modified Borg scale with the participants in a seated position. These assessments were conducted by a trained health professional following standardised recommendations [21, 22, 23].

### 2.5 The Main Outcome

The main outcome variable was the level of depression which was measured using the PHQ‐9, a nine‐item instrument based on Diagnostic and Statistical Manual criteria for major depressive disorders, and has been validated in the cardiac population [24]. Each item is rated on a 0–3 (0 = ‘not at all’ to 3 = ‘nearly every day’) scale, which relates to the frequency of the symptoms. This questionnaire is scored out of 27, with the depressive symptom severity categorised as minimal (0–4), mild [5, 6, 7, 8, 9], moderate [10, 11, 12, 13, 14], moderately severe [15, 16, 17, 18, 19] and severe [20, 21, 22, 23, 24, 25, 26, 27]. The scores were recategorised as ‘no depression’ for scores ≤ 9 and ‘depression’ for scores ≥ 10 based on its reported sensitivity and specificity of 88% in diagnosing major depressive disorder [25].

### 2.6 Description of the Cardiopulmonary Rehabilitation Programmes

On the first day of enrolment, all participants discussed with staff to set their goals for the programme and underwent individualised initial clinical, physical and psychosocial assessments by the cardiopulmonary rehabilitation team. The team comprised two part‐time nurses (registered and enrolled) with a combined equivalent of 42 h per week and a physiotherapist who works 2 h per week. The cardiac and pulmonary rehabilitation programmes at the Bathurst Health Service are designed as a 6‐ and 8‐week programme, respectively, where each participant attends one class (approximately 1 h) per week. Compared to the traditional structured programmes of 6–8 weeks in other regions, this short duration ‘half‐program’ was designed to accommodate the lack of qualified staff and other health professionals including exercise physiologists, dietitians and psychologists in this regional area.

Both programmes (cardiac and pulmonary rehabilitation) involve aerobic exercise training, strengthening exercise and education on strategies to control cardiovascular disease risk factors. Although there are variations in pace for the CR and PR programmes, their exercise routine is basically the same as per the recommendations of Heart Foundation Australia and Lung Foundation Australia [3, 4]. The aim of using the same exercises is to strengthen similar antigravity muscles and improve functional capacity, which is relevant for persons attending either CR or PR programme [26].

*The aerobic exercises* involve a 15‐min walking programme where participants get into a moderate—somewhat hard intensity (using the modified Borg scale) walk and another 15 min of either cycling, walking, or rowing at a moderate—somewhat hard intensity (using the modified Borg scale).

The muscular strengthening exercises are incorporated between two aerobic exercises and consist of a 15‐min routine targeting the upper limbs, lower limbs and core muscles. For the upper limbs, exercises include biceps curls using a 1 kg dumbbell in each hand (2 sets of 15 repetitions), progressing every 2 weeks by increasing the weight by 0.5 kg per hand and forward punches (based on participants' shoulder mobility) for 2 sets of 15 repetitions, progressing every 2 weeks by adding an extra set of 15 repetitions. For the lower limbs, exercises include sit‐to‐stand movements (without weights) for 2 sets of 15 repetitions, seated knee extensions (without weights) for 2 sets of 15 repetitions, seated heel raises for 2 sets of 15 repetitions and step‐ups and step‐downs using an aerobic stepper for 2 sets of 15 repetitions per limb. Each lower limb exercise progresses every 2 weeks by adding a 0.5 kg weight to each hand (for sit‐to‐stand and step‐ups) or each ankle (for knee extensions and heel raises).

Core strengthening includes high knee stepping in standing for 15 repetitions per limb, progressing every 2 weeks by adding an extra set of 15 repetitions. Balance exercises, performed within parallel bars for support, include tandem stance (each limb positioned in front for 30 s, 2 sets) and single leg stance (each limb raised for 30 s, 2 sets). These exercises progress every 2 weeks by adding an extra set of 30 s per limb. For the home programme, participants received an exercise sheet with two strengthening exercises for the upper and lower limbs and one balance exercise. These were adapted from their current routine in the programme to suit their home environment. These exercises are written with clear instructions following the Heart Foundation and Lung Foundation, Australia [2, 27].

### 2.7 Data Analysis

The data of all consenting participants who enrolled in the CR programme from January 2021 to December 2023 were included in this study. The normality of the data was tested using the Kolmogorov–Smirnov test and Lilliefors correction, with more than half of the variables identified not to be normally distributed. However, the application of the central limit theorem allowed the use of parametric statistical analysis for the data [28]. All results were presented as mean (standard deviation) or frequency (percentages). All analyses were two‐tailed, and *p* < 0.05 was considered statistically significant. Differences between pre‐ and post‐measurements of the mental (PHQ‐9) and physical (heart rate, blood pressure, oxygen saturation levels, waist circumference, HGS, rating of perceived exertion, 5‐STS and 6MWT) outcome variables of the participants were assessed using the paired *t*‐tests. Differences in depression scores between pre‐ and post‐measurements and rehabilitation programme types were determined using the Chi square test. One‐way analysis of variance (ANOVA) was used to determine the effect of the enrolment year on the main outcome variable. All analyses were conducted using the Statistical Package for the Social Sciences, version 29.0 (IBM Corp., Armonk, N.Y., USA).

## 3 Results

### 3.1 Demographic and Clinical Characteristics

Data from 205 adults who attended these programmes over the study period were available, but only the data for the 186 participants (90.7%) who completed the pre‐ and post‐rehabilitation assessments for the outcome measures were included in the analysis. Nineteen participants were excluded because they did not have complete demographic and/or clinical information. Of those included in the analysis, a summary statistic of their demographic and clinical characteristics is shown in Table 1. The characteristics of the participants included in this study were comparable with those of the excluded participants (*p* > 0.05) except for the 6MWT mean score.

**TABLE 1:** Demographic and clinical characteristics of participants.

Variables	Frequency, *n* (%)
Demography	
Sex, males *n* (%)	107 (57.5%)
Age (years)	69.34 (12.58)
Weight (kg)	87.87 (21.88)
Height (m)	1.69 (0.11)
Body mass index (kg/m^2^)	30.7 (8.32)
Year of enrolment	
2021/2022/2023	44 (23.7%)/57 (30.7%)/85 (45.7%)
Type of program, *n* (%)	
Cardiac/pulmonary	121 (65%)/65 (35%)
Baseline measures	
Heart rate (beats/min)	75.1 (13.5)
Waist circumference (cm)	104.7 (16.1)
5 Sit‐to‐Stand (seconds)	15.8 (7.4)
Six‐minute walk test (m)	328.6 (92.1)
Systolic blood pressure (mmHg)	133 (18)
Diastolic blood pressure (mmHg)	77 (10)
SPO_2_ (%)	96.2 (2.1)
Handgrip strength (*n* = 140)	
Right	29.9 (11.6)
Left	28.9 (11.3)
Borg Scale	0.31 (0.04)
Cardiac rehabilitation referring conditions, *n* (%)^a^	
Coronary artery disease/isolated CABG	30 (24.7%)/28 (23.1%)
Poor exercise tolerance/other cardiac surgeries	16 (13.2%)/9 (7.4%)
NSTEMI+coronary angiography/isolated AVR	7 (5.7%)/6 (4.9%)
Isolated MVR/cardiomyopathy	6 (4.9%)/5 (4.9%)
Chronic heart failure/CABG+valvular replacement	4 (3.3%)/3 (2.4%)
Not given/angina	3 (2.4%)/2 (1.6%)
Atrial fibrillation/infective carditis	2 (1.6%)/1 (0.8%)
Pulmonary rehabilitation referring conditions, *n* (%)^b^	
COPD/long COVID symptoms/poor exercise tolerance	26 (40.0%)/19 (29.3%)/8 (12.3%)
Shortness of breath/lobectomy/IPF	4 (6.2%)/3 (4.6%)/2 (3.1%)
ILD/pneumonia/not given	1 (1.5%)/1 (1.5%)/1 (1.5%)

*Note:* Data have been presented as a mean (standard deviation) otherwise frequency was used.

Abbreviations: AVR, Aortic valvular replacement; CABG, Coronary artery bypass graft; COPD, Chronic obstructive pulmonary disease; ILD, Interstitial lung disease; IPF, Idiopathic pulmonary fibrosis; MVR, Mitral valvular replacement; NSTEMI, Non‐ST‐elevation myocardial infarction; SPO2, Peripheral capillary oxygen saturation.

^a^ Data for 121 persons.

^b^ Data for 65 persons were used.

More than half of the participants were males (57.5%), with a higher mean BMI (30.7 ± 8.3 kg/m^2^) and the average age was 69 years. The average waist circumference was 104.7 (±16.1) cm. Sixty‐five per cent were attending the cardiac rehabilitation program. The primary diagnoses for participation for those in the CR programme were coronary artery disease and isolated CABG surgery and for those in the PR program, COPD and long COVID symptoms were their primary diagnoses (Table 1).

### 3.2 Mental and Physical Health Outcomes

**FIGURE 1:**
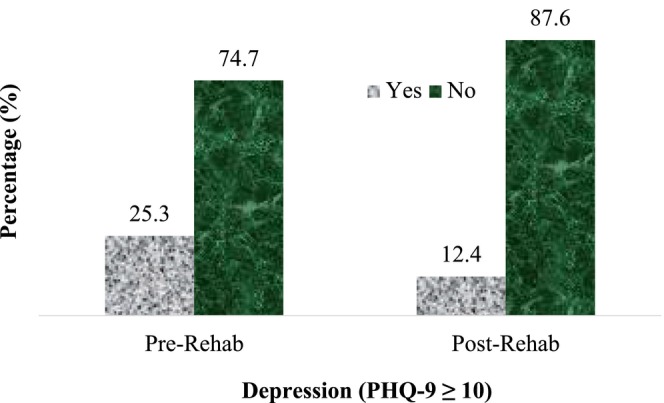
Comparison of pre‐ and post‐rehabilitation data according to the presence of depression.

Figure 1 presents the prevalence of depression in this study population and showed that 25.3% had major depressive disorder (PHQ‐9 ≥ 10) at the time of enrolment, which reduced by 50% at the completion of the programme (*p* < 0.05).

**FIGURE 2:**
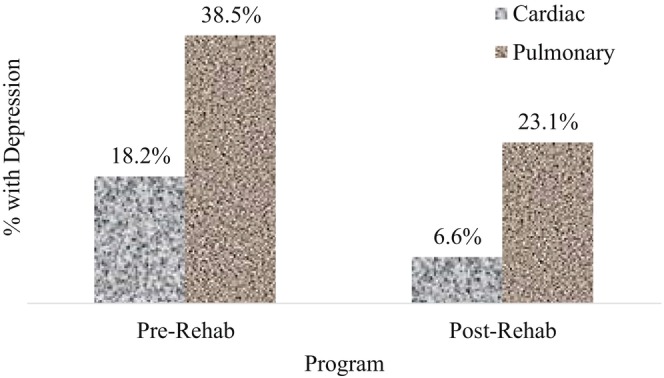
Comparison of the presence of depression across pre‐ and post‐rehabilitation data according to the type of programme attended.

Figure 2 shows that participants who attended PR had a higher prevalence of major depressive disorder than CR participants at enrolment. However, in both groups, significant reductions (*p* < 0.05) in major depressive disorder were observed post‐rehabilitation, with 11.6% and 15.4% reductions in the CR and PR participants, respectively (Figure 2).

Table 2 presents the mean differences (post minus pre) in measured variables for programme participants, while the absolute values (mean and standard deviation) are provided in Table S1. The enrolment years (2021, 2022 and 2023) had no significant influence on the measured variables (One‐way ANOVA, *p* > 0.05). Regarding the physical measures in all participants, and within the PR and CR groups, waist circumference, 5 Sit‐to‐Stand and 6MWT showed statistically significant changes post‐intervention. The time to complete the 5‐STS was reduced by 22% and 17% for participants in the CR and PR programmes, respectively, compared to pre‐rehabilitation values.

**TABLE 2:** Changes in physical outcome variables of the participants.

All participants (*n* = 186)	Mean difference (95% CI)	*p*	Change (%)
Heart rate (beats/min)	1.48 (−0.16, 3.12)	0.076	−1.99
Waist circumference (cm)	0.79 (0.27, 1.31)	**0.003**	−0.75
5 Sit‐to‐stand (seconds)	3.21 (2.33, 4.10)	**< 0.001**	−20.83
Six‐minute walk test (m)	−49.27 (−57.53, −41.01)	**< 0.001**	14.9
Systolic blood pressure (mmHg)	2.23 (−0.20, 4.82)	0.071	−1.73
Diastolic blood pressure (mmHg)	1.14 (−0.21, 2.49)	0.098	−1.49
SPO_2_	−0.12 (−0.47, 0.24)	0.510	0.12
2021 Enrolment	44 (23.7%)		
2022 Enrolment	57 (30.7%)		
2023 Enrolment	85 (45.7%)		
Cardiac rehabilitation participants (*n* = 121)			
Heart rate (beats/min)	1.88 (−0.06, 0.38)	0.058	−2.60
Waist circumference (cm)	0.66 (0.02, 1.31)	**0.044**	−0.63
5 Sit‐to‐Stand (seconds)	3.42 (2.29, 4.55)	**< 0.001**	−22.09
Six‐minute walk test (m)	−52. 41 (−62.53, −42.30)	**< 0.001**	15.47
Systolic blood pressure (mmHg)	2.07 (0.10, 5.14)	0.183	−1.58
Diastolic blood pressure (mmHg)	0.88 (−0.83, 2.60)	0.309	−1.16
SPO_2_	0.05 (−0.28, 0.38)	0.766	−0.05
2021 Enrolment	25 (20.7%)		
2022 Enrolment	38 (31.4%)		
2023 Enrolment	58 (47.9%)		
Pulmonary rehabilitation participants (*n* = 65)			
Heart rate (beats/min)	0.75 (−2.31, 3.82)	0.625	−0.95
Waist circumference (cm)	1.02 (0.14, 1.91)	**0.024**	−0.96
5 Sit‐to‐stand (seconds)	2.83 (1.39, 4.28)	**< 0.001**	−17.32
Six‐minute walk test (m)	−43.42 (−57.98, −28.85)	**< 0.001**	14.01
Systolic blood pressure (mmHg)	2.75 (−1.71, 7.21)	0.222	−2.00
Diastolic blood pressure (mmHg)	1.62 (−0.64, 3.87)	0.157	−2.09
SPO_2_	−0.43 (−1.25, 0.38)	0.295	0.45
2021 Enrolment	19 (29.2%)		
2022 Enrolment	19 (29.2%)		
2023 Enrolment	27 (41.6%)		

*Note:* All changes in the outcome variables were presented as mean difference (95% confidence interval) except for the enrolment year, which was presented as frequency (%). Bolded are significant *p*‐values.

## 4 Discussion

The findings of this study showed the significant impact of a short rehabilitation programme in regional NSW. Participants attending cardiac or pulmonary rehabilitation programmes significantly improved their mental and physical health, especially depressive symptoms, time to complete 5‐STS, 6MWT distance and waist circumference. In contrast, blood pressure, heart rate and blood oxygen saturation levels were similar between pre‐ and post‐rehabilitation assessments. Despite the progressive increase in the number of enrolments since 2021 following the COVID‐19 pandemic, the year of enrolment was not identified as a significant contributor to these pre‐ and post‐rehabilitation changes identified in the improved variables. Overall, the current study showed that in regional and rural settings, where there is a limited number of health professionals to conduct standard, twice to thrice per week CR rehabilitation programmes, attending a 1‐day CR programme weekly is still adequate to elicit improvements in the level of depression and physical function of an individual.

Given the increasing prevalence of depression among patients living with cardiac and respiratory diseases, attending rehabilitation programmes has been shown to have beneficial effects on a patient's mental well‐being [29, 30]. The findings of a decrease in the proportion experiencing major depression post‐rehabilitation are congruent with previous studies conducted among individuals who attended CR or PR programmes [30, 31, 32, 33] elsewhere. This improvement could be partly attributed to the social interaction of participating in cardiopulmonary rehabilitation, where participants interact, support each other and recount their personal experiences, potentially serving as a means to alleviate apprehension among participants [34]. Further, this improvement could be due to the reported anti‐depressant effects of exercises in reshaping the brain structure, promoting behavioural adaptation changes, and maintaining the integrity of the hippocampus and white matter of the brain [35]. These neurophysiological changes enhance the nervous system processing and delay cognitive deterioration in depressed patients [36].

This study found an improvement in the time to complete the 5‐STS test, which was similar to previous studies [37, 38] that reported stronger lower limb muscles, improved balance and low falls risk among participants who attended cardiorespiratory rehabilitation [39, 40]. This is explained by the impact of cardiac and respiratory diseases in causing reduced involvement in physical activity, with concomitant alterations in skeletal muscle metabolism, systemic inflammation and decreased muscle capillary density, which leads to generalised muscle atrophy [41]. Further, these processes are accelerated by the possible sarcopenic profile of these patients, as previous studies have reported positive associations between sarcopenia and cardiac or pulmonary diseases [42, 43]. This explains why one of the core components of cardiac and pulmonary rehabilitation programmes is graded strengthening exercises of the limbs. Improvement in lower limb muscle strength whilst maintaining optimal balance requires the coordination of several lower limb and trunk muscle groups and thus is an essential component of independence [37].

Another significant finding of this study was the reduction in waist circumference post‐rehabilitation programme. Rehabilitation programmes often incorporate physical activity components, such as cardiovascular exercise and strength training, which can contribute to overall weight loss and reductions in abdominal fat, including waist circumference. Also, improvements in dietary habits and lifestyle behaviours, commonly addressed in rehabilitation programmes, may contribute to reductions in waist circumference. Participants may adopt healthier eating patterns and decrease their intake of calorie‐dense foods, leading to reductions in abdominal fat. This finding aligns with previous work by Greenwood et al. [44] and Silva et al. [45] demonstrating the effectiveness of rehabilitation programmes in reducing waist circumference. In that study [44], the authors found that exercise‐based rehabilitation programmes were associated with significant reductions in waist circumference among individuals with CKD. Silva et al. [45] reported significant reductions in waist circumference following a multidisciplinary rehabilitation programme for individuals with obesity. Overall, the reduction in waist circumference observed post‐rehabilitation programme likely reflects the multifaceted approach of these interventions, which target both physical activity and dietary behaviours to promote weight loss and improvements in body composition. Further research is warranted to elucidate the specific mechanisms underlying these effects and to optimise the design and implementation of rehabilitation programmes for individuals seeking to reduce abdominal fat and improve overall health.

### 4.1 Limitations and Strengths of the Study

The study has several limitations to consider. First, there was no control group, which prevents direct comparison between participants attending rehabilitation programmes and those not participating, potentially affecting the attribution of observed improvements solely to the programmes. Second, the small sample size and voluntary enrolment in cardiac or pulmonary rehabilitation programmes may introduce selection bias, with enrolled individuals possibly more motivated to improve their health outcomes, thus possibly overestimating programme effectiveness. Third, the study was conducted in a regional and rural setting, which may limit the generalisability of the findings to other settings with better access to healthcare services and resources. Fourth, the lack of longer‐term follow‐up data prevents insights into the sustainability of observed improvements over time and the potential need for ongoing support or intervention. Lastly, while the study assessed several outcome measures, important variables such as quality of life and medication adherence were not included, potentially limiting the comprehensive understanding of programme impact. Despite these limitations, the study contributes valuable insights into the effectiveness of cardiac and pulmonary rehabilitation programmes in improving mental well‐being and physical function among participants in regional and rural settings. The significant improvements observed in most of the measured outcomes, especially waist circumference, after attending just one session per week for only 6 weeks underscore the importance of such programmes, particularly in rural settings with limited options. Future research with larger sample sizes, control groups, longer follow‐up periods and comprehensive outcome measures is needed to further confirm the benefits of these programmes and address the identified limitations. Additionally, future studies may look at assessing the balance of the participants, particularly since the majority were older individuals; their lived experiences and barriers to attending cardiorespiratory rehabilitation may provide further insights.

### 4.2 Clinical Implications

From a clinical perspective, attending even one session per week of cardiac or pulmonary rehabilitation can yield positive outcomes for participants, particularly in settings where resources are scarce. Healthcare providers, especially those working in regional and rural areas, should consider incorporating and promoting rehabilitation programmes as part of comprehensive care plans for patients with cardiac and respiratory diseases. Furthermore, the findings underscore the need for funding for ongoing support and intervention beyond the immediate post‐rehabilitation period to sustain the observed improvements in mental well‐being and physical function. Overall, this contributes to the growing body of evidence supporting the benefits of cardiac and pulmonary rehabilitation programmes in improving health outcomes for individuals living with cardiovascular and respiratory conditions. Future research with larger sample sizes and longer follow‐up periods is warranted to better understand the long‐term effects of these programmes. By addressing these gaps in knowledge, healthcare providers can better tailor rehabilitation interventions to meet the needs of patients in regional and rural settings, ultimately improving overall health and well‐being.

## 5 Conclusion

The findings of this study underscore the important role of cardiac and pulmonary rehabilitation programmes, particularly in addressing the healthcare disparities prevalent in rural and regional areas characterised by limited‐service access. Our findings highlight the significant impact of even short‐term participation in these programmes on enhancing both the mental well‐being and physical function of the participants. Notably, the observed improvements in key measures, including the reduction in the proportion of participants experiencing major depression, enhanced mobility as evidenced by decreased time to complete the 5‐STS, increased 6MWT distance and reduction in waist circumference, underscore the importance of implementing and advocating for the expansion of such rehabilitation initiatives in underserved regions. This is particularly crucial as rural and regional communities grapple with persistent healthcare resource shortages; therefore, securing funding for sustainable programmes led by allied health professionals becomes imperative. Programmes such as this do not only address immediate healthcare needs of people in their community but lay the foundation for positive long‐term health and general well‐being outcomes, thereby mitigating the impact of service deficiencies on vulnerable populations. Investing in and prioritising the dissemination of rehabilitation services in these areas becomes essential steps towards fostering equitable access to quality healthcare for all.

## Author Contributions

**Nnamdi Mgbemena:** conceptualization, writing – review and editing, writing – original draft, formal analysis, methodology. **Jane Thompson:** conceptualization, writing – original draft, writing – review and editing, investigation, resources, methodology. **Uchechukwu Levi Osuagwu:** conceptualization, methodology, writing – review and editing, writing – original draft, formal analysis.

## Disclosure

The authors have nothing to report.

## Ethics Statement

The study followed the tenets of the Declaration of Helsinki for human subjects (as revised in Brazil 2013). Prior to data collection, ethics approval for the study was obtained from Western New South Wales Local Health District; Greater Western Human Research Ethics Committee, University Research Ethics Committee (2021/ETH00556).

## Conflicts of Interest

The authors declare no conflicts of interest.

## Supporting information


Appendix S1.

## Data Availability

The data that support the findings of this study are available on request from the corresponding author. The data are not publicly available due to privacy or ethical restrictions.

## References

[1] M. D. Hasnain , D. Patrick , and S. T. Rod , “Cardiac rehabilitation,” BMJ [British Medical Journal] 351 (2015): h5000.26419744

[2] J. A. Alison , Z. J. McKeough , K. Johnston , et al., “Australian and New Zealand Pulmonary Rehabilitation Guidelines,” Respirology 22, no. 4 (2017): 800–819, 10.1111/resp.13025. 28339144

[3] Lung Foundation Australia , “Pulmonary Rehabilitation Toolkit 2023,” accessed March 25, 2024, https://lungfoundation.com.au/health‐professionals/clinical‐information/pulmonary‐rehabilitation/pr‐toolkit/.

[4] Heart Foundation , “Cardiac Rehabilitation. Supporting Your Recovery 2020,” accessed March 25, 2024, https://assets.contentstack.io/v3/assets/blt8a393bb3b76c0ede/blta676a981e19696d6/659cb867bb2e10cf2c012952/Cardiac‐Rehab_Brochure_2020.pdf.

[5] R. S. Taylor , H. M. Dalal , and S. T. J. McDonagh , “The Role of Cardiac Rehabilitation in Improving Cardiovascular Outcomes,” Nature Reviews Cardiology 19, no. 3 (2022): 180–194. 34531576 10.1038/s41569-021-00611-7 PMC8445013

[6] Australian Institute of Health and Welfare , “Treatment Pathways for People Hospitalised for Acute Coronary Syndrome, Australia,” 2024, accessed March 27, 2024, https://www.aihw.gov.au/reports/heart‐stroke‐vascular‐diseases/treatment‐pathways‐for‐people‐hospitalised‐for‐acu/contents/background.

[7] Heart Foundation , “Acute Coronary Syndromes (ACS) Clinical Guidelines,” 2024 accessed January 21, 2025, https://www.heartfoundation.org.au/for‐professionals/fp‐acs‐guidelines.

[8] S. Sritharan , B. Wilsmore , J. Wiggers , et al., “Rural‐Urban Differences in Outcomes of Acute Cardiac Admissions in a Large Health Service,” JACC: Advances 3, no. 11 (2024): 101328, 10.1016/j.jacadv.2024.101328. 39469611 PMC11513678

[9] E. De Gruyter , G. Ford , and B. Stavreski , “Economic and Social Impact of Increasing Uptake of Cardiac Rehabilitation Services—A Cost Benefit Analysis,” Heart, Lung & Circulation 25, no. 2 (2016): 175–183. 26442971 10.1016/j.hlc.2015.08.007

[10] P. E. Field , R. C. Franklin , R. N. Barker , I. Ring , and P. A. Leggat , “Cardiac Rehabilitation Services for People in Rural and Remote Areas: An Integrative Literature Review,” Rural and Remote Health 18, no. 4 (2018): 1–3. 10.22605/RRH4738 30403491

[11] Australian Institute of Health and Welfare , “Chronic Respiratory Conditions,” 2024, accessed January 21, 2025, https://www.aihw.gov.au/reports/chronic‐respiratory‐conditions/chronic‐respiratory‐conditions/contents/about.

[12] L. Desveaux , T. Janaudis‐Ferreira , R. Goldstein , and D. Brooks , “An International Comparison of Pulmonary Rehabilitation: A Systematic Review,” COPD: Journal of Chronic Obstructive Pulmonary Disease 12, no. 2 (2015): 144–153. 24984085 10.3109/15412555.2014.922066

[13] J. L. Cousins , R. Wood‐Baker , P. A. B. Wark , et al., “Management of Acute COPD Exacerbations in Australia: Do We Follow the Guidelines?,” ERJ Open Research 6, no. 2 (2020): 270–2019. 10.1183/23120541.00270-2019 PMC7167211 32337215

[14] K. Johnston , M. Young , K. Grimmer , R. Antic , and P. Frith , “Frequency of Referral to and Attendance at a Pulmonary Rehabilitation Programme Amongst Patients Admitted to a Tertiary Hospital With Chronic Obstructive Pulmonary Disease,” Respirology 18, no. 7 (2013): 1089–1094. 23711304 10.1111/resp.12128

[15] K. N. Johnston , M. Young , K. A. Grimmer , R. Antic , and P. A. Frith , “Barriers to, and Facilitators for, Referral to Pulmonary Rehabilitation in COPD Patients From the Perspective of Australian General Practitioners: A Qualitative Study,” Primary Care Respiratory Journal 22, no. 3 (2013): 319–324. 10.4104/pcrj.2013.00062 PMC6442818 23797679

[16] J. S. Watson , R. E. Jordan , P. Adab , I. Vlaev , A. Enocson , and S. Greenfield , “Investigating Primary Healthcare practitioners' Barriers and Enablers to Referral of Patients With COPD to Pulmonary Rehabilitation: A Mixed‐Methods Study Using the Theoretical Domains Framework,” BMJ Open 12, no. 1 (2022): e046875. 10.1136/bmjopen-2020-046875 PMC8772414 35045995

[17] C. L. Johnston , L. J. Maxwell , G. P. Maguire , and J. A. Alison , “Does Delivery of a Training Program for Healthcare Professionals Increase Access to Pulmonary Rehabilitation and Improve Outcomes for People With Chronic Lung Disease in Rural and Remote Australia?,” Australian Health Review 38, no. 4 (2014): 387–395. 25030042 10.1071/AH14009

[18] World Health Organization , “Chronic Obstructive Pulmonary Disease,” 2023 accessed March 27, 2024, https://www.who.int/news‐room/fact‐sheets/detail/chronic‐obstructive‐pulmonary‐disease‐(copd).

[19] World Health Organization , “Cardiovascular Diseases,” 2021 accessed March 27, 2024, https://www.who.int/news‐room/fact‐sheets/detail/cardiovascular‐diseases‐(cvds).

[20] Heart Foundation , “A Pathway to Cardiac Recovery Standardised Program Content for Phase II Cardiac Rehabilitation,” 2019 accessed March 27, 2024, https://www.heartfoundation.org.au/for‐professionals/cardiac‐rehabilitation.

[21] R. Ross , I. J. Neeland , S. Yamashita , et al., “Waist Circumference as a Vital Sign in Clinical Practice: A Consensus Statement From the IAS and ICCR Working Group on Visceral Obesity,” Nature Reviews. Endocrinology 16, no. 3 (2020): 177–189. 10.1038/s41574-019-0310-7 PMC7027970 32020062

[22] N. Mgbemena , A. Jones , P. Saxena , N. Ang , S. Senthuran , and A. Leicht , “Acute Changes in Handgrip Strength, Lung Function and Health‐Related Quality of Life Following Cardiac Surgery,” PLoS One 17, no. 2 (2022): e0263683. 35196327 10.1371/journal.pone.0263683 PMC8865673

[23] E. J. Kammin , “The 6‐Minute Walk Test: Indications and Guidelines for Use in Outpatient Practices,” Journal for Nurse Practitioners 18, no. 6 (2022): 608–610. 10.1016/j.nurpra.2022.04.013 PMC9095083 35578650

[24] Heart Foundation , “Depression in Patients With Coronary Heart Disease. A Practical Tool for Screening Your Patients, Australia,” 2013 accessed March 27, 2024, https://assets.contentstack.io/v3/assets/blt8a393bb3b76c0ede/blt2889cbeb7f3fae28/65b073c77a1dd75d3ce156cb/01_Depression‐screening‐support‐tool.PDF.

[25] K. Kroenke , R. L. Spitzer , and J. B. W. Williams , “The PHQ‐9,” Journal of General Internal Medicine 16, no. 9 (2001): 606–613. 11556941 10.1046/j.1525-1497.2001.016009606.x PMC1495268

[26] Z. Louvaris and I. Vogiatzis , “Physiological Basis of Cardiopulmonary Rehabilitation in Patients With Lung or Heart Disease,” Breathe (Sheffield, England) 11, no. 2 (2015): 120–127. 26306112 10.1183/20734735.021114 PMC4487369

[27] S. Woodruffe , L. Neubeck , R. A. Clark , et al., “Australian Cardiovascular Health and Rehabilitation Association (ACRA) Core Components of Cardiovascular Disease Secondary Prevention and Cardiac Rehabilitation 2014,” Heart, Lung & Circulation 24, no. 5 (2015): 430–441. 25637253 10.1016/j.hlc.2014.12.008

[28] S. G. Kwak and J. H. Kim , “Central Limit Theorem: The Cornerstone of Modern Statistics,” Korean Journal of Anesthesiology 70, no. 2 (2017): 144–156. 28367284 10.4097/kjae.2017.70.2.144 PMC5370305

[29] A. S. Iyer , K. E. Holm , S. P. Bhatt , et al., “Symptoms of Anxiety and Depression and Use of Anxiolytic‐Hypnotics and Antidepressants in Current and Former Smokers With and Without COPD—A Cross Sectional Analysis of the COPDGene Cohort,” Journal of Psychosomatic Research 118 (2019): 18–26. 30782350 10.1016/j.jpsychores.2019.01.002 PMC6383809

[30] F. J. Penedo and J. R. Dahn , “Exercise and Well‐Being: A Review of Mental and Physical Health Benefits Associated With Physical Activity,” Current Opinion in Psychiatry 18, no. 2 (2005): 189–193. 16639173 10.1097/00001504-200503000-00013

[31] M. Roy and M. Benzo , eds., Improving Depression in COPD Patients Through Home‐Based Pulmonary Rehabilitation With Health Coaching (American Thoracic Society International Conference, 2023).

[32] J. Quindry , M. McNamara , C. Oser , and C. Fogle , “Assessment of Clinical Depression Metrics in Cardiac Patients Using the Patient Health Questionnaire‐9 Before and After Phase‐II Cardiac Rehabilitation,” Sports Medicine and Health Science 6, no. 3 (2023): 240–245. 39234489 10.1016/j.smhs.2023.09.004 PMC11369830

[33] K. M. McKenzie , L. K. Park , E. J. Lenze , et al., “A Prospective Cohort Study of the Impact of Outpatient Intensive Cardiac Rehabilitation on Depression and Cardiac Self‐Efficacy,” American Heart Journal Plus 13 (2022): 100100. 36407054 10.1016/j.ahjo.2022.100100 PMC9671388

[34] M. J. Blikman , H. R. Jacobsen , G. E. Eide , and E. Meland , “How Important Are Social Support, Expectations and Coping Patterns During Cardiac Rehabilitation,” Rehabilitation Research and Practice 2014 (2014): 973549. 25302122 10.1155/2014/973549 PMC4180384

[35] P. E. Vasques , H. Moraes , H. Silveira , A. C. Deslandes , and J. Laks , “Acute Exercise Improves Cognition in the Depressed Elderly: The Effect of Dual‐Tasks,” Clinics (São Paulo, Brazil) 66, no. 9 (2011): 1553–1557. 22179158 10.1590/S1807-59322011000900008 PMC3164403

[36] J. L. Zhao , W. T. Jiang , X. Wang , Z. D. Cai , Z. H. Liu , and G. R. Liu , “Exercise, Brain Plasticity, and Depression,” CNS Neuroscience & Therapeutics 26, no. 9 (2020): 885–895. 32491278 10.1111/cns.13385 PMC7415205

[37] M. L. Puthoff and D. Saskowski , “Reliability and Responsiveness of Gait Speed, Five Times Sit to Stand, and Hand Grip Strength for Patients in Cardiac Rehabilitation,” Cardiopulmonary Physical Therapy Journal 24, no. 1 (2013): 31–37. 23754937 PMC3677181

[38] E. Zampogna , P. Pignatti , N. Ambrosino , et al., “The 5‐Repetition Sit‐To‐Stand Test as an Outcome Measure for Pulmonary Rehabilitation in Subjects With Asthma,” Respiratory Care 66, no. 5 (2021): 769–776. 33593936 10.4187/respcare.08452

[39] S. Buatois , C. Perret‐Guillaume , R. Gueguen , et al., “A Simple Clinical Scale to Stratify Risk of Recurrent Falls in Community‐Dwelling Adults Aged 65 Years and Older,” Physical Therapy 90, no. 4 (2010): 550–560. 20203094 10.2522/ptj.20090158

[40] R. W. Bohannon , D. J. Bubela , S. R. Magasi , Y. C. Wang , and R. C. Gershon , “Sit‐To‐Stand Test: Performance and Determinants Across the Age‐Span,” Isokinetics and Exercise Science 18, no. 4 (2010): 235–240. 25598584 10.3233/IES-2010-0389 PMC4293702

[41] P. J. Kennel , D. M. Mancini , and P. C. Schulze , “Skeletal Muscle Changes in Chronic Cardiac Disease and Failure,” Comprehensive Physiology 5, no. 4 (2015): 1947–1969. 26426472 10.1002/cphy.c110003 PMC6752037

[42] N. He , Y. Zhang , L. Zhang , S. Zhang , and H. Ye , “Relationship Between Sarcopenia and Cardiovascular Diseases in the Elderly: An Overview,” Frontiers in Cardiovascular Medicine 8 (2021): 743710. 34957238 10.3389/fcvm.2021.743710 PMC8695853

[43] N. Martínez‐Luna , A. Orea‐Tejeda , D. González‐Islas , et al., “Association Between Body Composition, Sarcopenia and Pulmonary Function in Chronic Obstructive Pulmonary Disease,” BMC Pulmonary Medicine 22, no. 1 (2022): 106. 35346135 10.1186/s12890-022-01907-1 PMC8962175

[44] S. A. Greenwood , P. Koufaki , T. H. Mercer , et al., “Effect of Exercise Training on Estimated GFR, Vascular Health, and Cardiorespiratory Fitness in Patients With CKD: A Pilot Randomized Controlled Trial,” American Journal of Kidney Diseases 65, no. 3 (2015): 425–434, 10.1053/j.ajkd.2014.07.015. 25236582

[45] G. Silva , M. Ghiraldi , I. Santos , et al., “Effects of a Multidisciplinary Approach on the Anthropometric and Body Composition Responses of Obese Adolescents (Efectos de Un Abordaje Multidisciplinario Sobre Las Respuestas antropométricas y de composición Corporal de Adolescentes Obesos),” Retos 46 (2022): 323–329, 10.47197/retos.v46.93066.

