# Supplementary material for: Changes in Mental and Physical Health Outcomes Following One Day a Week Cardiopulmonary Rehabilitation in Regional New South Wales
Source: Aust J Rural Health. 2025 Mar 24;33(2):e70033. doi: 10.1111/ajr.70033 (PMC11931675; doi:10.1111/ajr.70033)
Supplement: Supplementary file 1 — Appendix S1. [file AJR-33-0-s001.docx]

**Supplemental Table 1: Pre- and post- rehabilitation assessments (including their mean differences) of the participants**

| **All participants**  **(n = 186)** | **Pre -rehabilitation**  **[Mean (SD)]** | **Post-rehabilitation**  **[Mean (SD)]** | **Mean Difference (95% CI)** | **p-value** | **Change %** |
| --- | --- | --- | --- | --- | --- |
| Heart rate (beats/minute) | 75.05 (13.48) | 73.56 (12.41) | 1.48 (-0.16, 3.12) | 0.076 | -1.99 |
| Waist circumference (cm) | 104.71 (16.05) | 103.92 (15.91) | 0.79 (0.27, 1.31) | 0.003 | -0.75 |
| 5 Sit-to Stand (seconds) | 15.80 (7.39) | 12.58 (4.46) | 3.21 (2.33, 4.10) | <0.001 | -20.83 |
| PHQ-9 | 6.35 (4.87) | 4.22 (4.05) | 2.13 (1.60, 2.67) | <0.001 | -33.54 |
| Six-minute walk test (m) | 328.60 (92.10) | 377.87 (99.95) | -49.27 (-57.53, -41.01) | <0.001 | 14.9 |
| Systolic blood pressure (mmHg) | 133.19 (18.18) | 130.88 (17.05) | 2.23 (-0.20, 4.82) | 0.071 | -1.73 |
| Diastolic blood pressure (mmHg) | 76.59 (10.04) | 75.45 (8.95) | 1.14 (-0.21, 2.49) | 0.098 | -1.49 |
| SPO_2_ | 96.17 (2.11) | 96.29 (2.22) | -0.12 (-0.47, 0.24) | 0.510 | 0.12 |
| **Cardiac rehabilitation participants (n = 121)** |  |  |  |  |  |
| Heart rate (beats/minute) | 72.24 (12.25) | 70.36 (11.49) | 1.88 (-0.06, 0.38) | 0.058 | -2.60 |
| Waist circumference (cm) | 104.01 (15.37) | 103.35 (15.23) | 0.66 (0.02, 1.31) | 0.044 | -0.63 |
| 5 Sit-to Stand (seconds) | 15.48 (7.08) | 12.06 (4.16) | 3.42 (2.29, 4.55) | <0.001 | -22.09 |
| PHQ-9 | 5.55 (4.41) | 3.22 (3.23) | 2.33 (1.69, 2.97) | <0.001 | -41.98 |
| Six-minute walk test (m) | 338.74 (87.74) | 391.15 (96.65) | -52.41 (-62.53, -42.30) | <0.001 | 15.47 |
| Systolic blood pressure (mmHg) | 130.93 (18.67) | 128.86 (17.79) | 2.07 (0.099, 5.14) | 0.183 | -1.58 |
| Diastolic blood pressure (mmHg) | 76.04 (10.23) | 75.16 (9.08) | 0.88 (-0.83, 2.60) | 0.309 | -1.16 |
| SPO_2_ | 96.68 (1.46) | 96.63 (1.48) | 0.05 (-0.28, 0.38) | 0.766 | -0.05 |
| **Pulmonary Rehabilitation participants (n = 65)** |  |  |  |  |  |
| Heart rate (beats/minute) | 80.28 (14.18) | 79.52 (11.99) | 0.75 (-2.31, 3.82) | 0.625 | -0.95 |
| Waist circumference (cm) | 106.00 ()17.30 | 104.98 (17.17) | 1.02 (0.14, 1.91) | 0.024 | -0.96 |
| 5 Sit-to Stand (seconds) | 16.40 (7.96) | 13.56 (4.83) | 2.83 (1.39, 4.28) | <0.001 | -17.32 |
| PHQ-9 | 7.83 (5.35) | 6.06 (4.75) | 1.77 (0.77, 2.77) | <0.001 | -22.61 |
| Six-minute walk test (m) | 309.74 (97.60) | 353.15 (102.02) | -43.42 (-57.98, -28.85) | <0.001 | 14.01 |
| Systolic blood pressure (mmHg) | 137.40 (16.53) | 134.65 (14.87) | 2.75 (-1.71, 7.21) | 0.222 | -2.00 |
| Diastolic blood pressure (mmHg) | 77.60 (9.67) | 75.98 (8.75) | 1.62 (-0.64, 3.87) | 0.157 | -2.09 |
| SPO_2_ | 95.23 (2.74) | 95.66 (3.09) | -0.43 (-1.25, 0.38) | 0.295 | 0.45 |

PHQ-9 - patient health questionnaire-9; SPO_2_ – Peripheral capillary oxygen saturation
